# Peripheral nervous system safety signals of antibody–drug conjugates: cross-database reproducibility and labeling gaps identified using FAERS, JADER, and CVARD

**DOI:** 10.3389/fphar.2026.1786431

**Published:** 2026-04-21

**Authors:** Xiaojie Feng, Yabin Qin, Hongqing Ma, Rui Feng, Liman Huo

**Affiliations:** 1 Department of Pharmacy, The Fourth Hospital of Hebei Medical University, Shijiazhuang, China; 2 Department of Pharmacy, Hebei Children’s Hospital, Shijiazhuang, Hebei, China

**Keywords:** antibody–drug conjugates, cross-database reproducibility, drug safety, FAERS, peripheral nervous system, pharmacovigilance, regulatory labeling, spontaneous reporting systems

## Abstract

**Objective:**

To characterize the spectrum, strength, and cross-database reproducibility of peripheral nervous system (PNS) safety signals associated with antibody–drug conjugates (ADCs), and to examine discrepancies between post-marketing pharmacovigilance signals and current regulatory labeling.

**Methods:**

A multi-database pharmacovigilance study was conducted using spontaneous adverse event reports from the FDA Adverse Event Reporting System (FAERS; Q1-2025Q3), the Japanese Adverse Drug Event Report (JADER; 2004Q1–2025 Q3), and the Canada Vigilance Adverse Reaction Database (CVARD; January 1965 - October 2025). PNS-related adverse events were identified using the MedDRA Standardised MedDRA Query “Peripheral neuropathy,” supplemented by expert-curated preferred terms. Disproportionality analyses were performed using the reporting odds ratio, proportional reporting ratio, empirical Bayes geometric mean, and information component. Safety signals detected in FAERS were evaluated for reproducibility in JADER and CVARD and compared with PNS events described in current prescribing information.

**Results:**

Overall, 31,592 post-marketing reports in which an ADC was recorded as the primary suspect were identified in FAERS, of which 1,395 involved PNS-related adverse events. A broad spectrum of PNS toxicities was observed, with peripheral neuropathy, polyneuropathy, peripheral sensory neuropathy, and peripheral sensorimotor neuropathy being the most frequently reported phenotypes. Consistent and robust associations were observed across all three databases, with reporting odds ratios of 4.62 (95% confidence interval [CI] 4.39–4.87) in FAERS, 42.84 (95% CI 39.56–46.40) in JADER, and 20.79 (95% CI 18.12–23.85) in CVARD. Rare but clinically severe neuropathic conditions, including Guillain–Barré syndrome and chronic inflammatory demyelinating polyradiculoneuropathy, also met predefined signal-detection criteria. Time-to-onset analyses demonstrated substantial heterogeneity, with median onset occurring within approximately 20–40 days for several ADCs, whereas delayed presentations beyond 80–100 days were observed for others. A large proportion of PNS reports were classified as serious, most commonly involving hospitalization. Notably, several PNS signals identified through post-marketing surveillance were not consistently described in current prescribing information.

**Conclusion:**

This multi-database pharmacovigilance analysis identified robust and reproducible PNS safety signals associated with ADCs, including clinically relevant events not uniformly described in current regulatory labeling. These findings are hypothesis-generating and support signal prioritization rather than causal inference, underscoring the importance of ongoing post-marketing surveillance to inform regulatory awareness and risk communication as ADC use continues to expand.

## 1 Introduction

Antibody–drug conjugates (ADCs) have become an increasingly important class of anticancer therapeutics by combining the target specificity of monoclonal antibodies with the cytotoxic potency of small-molecule payloads (Beck et al., 2017; Drago et al., 2021). Since 2019, the number of ADCs approved by the U.S. Food and Drug Administration (FDA) has expanded rapidly, with indications extending across both solid tumors and hematologic malignancies and increasing use in earlier lines of therapy (Khongorzul et al., 2020). As clinical exposure to ADCs continues to grow, systematic post-marketing characterization of ADC-associated toxicities has become a key pharmacovigilance priority.

Among reported toxicities, peripheral nervous system (PNS) adverse events—including peripheral neuropathy, sensorimotor dysfunction, and immune-mediated neuropathies—are of particular clinical relevance, as they may lead to dose modification, treatment discontinuation, or persistent functional impairment, thereby compromising therapeutic benefit and patient quality of life (Ogitani et al., 2016a; Staff et al., 2017; Seretny et al., 2014). PNS toxicity is well recognized for certain ADCs, especially those incorporating microtubule-disrupting payloads such as monomethyl auristatin E (MMAE) or related auristatins (Sco and tt, 2017; Kung Sutherland et al., 2013). However, the overall spectrum of PNS-related adverse events across the expanding ADC class remains incompletely characterized.

Evidence from pre-approval clinical trials is limited by selective eligibility criteria, relatively short follow-up, and protocol-driven monitoring, which may underestimate the frequency, heterogeneity, and temporal patterns of neuropathic events encountered in routine clinical practice (Paller et al., 2014; Gyawali and Naci, 2021). In addition, regulatory prescribing information (PI) often describes neuropathy using broad categories and may not capture less common, delayed-onset, or clinically distinct PNS phenotypes, raising concerns regarding potential under-recognition of clinically meaningful neurotoxicity signals.

Spontaneous reporting systems (SRSs) provide an essential complement to clinical trials by enabling post-marketing detection of rare, serious, or unexpected adverse events at a population level (Hauben and Aronson, 2009; Hazell and Shakir, 2006). However, most published pharmacovigilance studies assessing ADC-associated neuropathy have relied exclusively on the FDA Adverse Event Reporting System (FAERS) and applied a limited number of disproportionality methods, potentially constraining signal robustness and external validity (Tang et al., 2024; Bate and Evans, 2009). Systematic cross-validation of PNS-related signals across independent national SRSs and structured comparisons with regulatory labeling therefore remain limited.

To address these gaps, we conducted a multi-database pharmacovigilance study using FAERS, the Japanese Adverse Drug Event Report (JADER) database, and the Canada Vigilance Adverse Reaction Database (CVARD). All FDA-approved ADCs available up to October 2025 were evaluated for associations with PNS-related adverse events using four complementary disproportionality algorithms—reporting odds ratio (ROR), proportional reporting ratio (PRR), empirical Bayes geometric mean (EBGM), and information component (IC). PNS preferred terms were identified using the Standardised MedDRA Query “Peripheral neuropathy” and expanded through expert clinical curation. We further assessed cross-database reproducibility of FAERS-identified signals and examined concordance between post-marketing findings and PNS events documented in current prescribing information.

Through this approach, the present study provides a comprehensive post-marketing evaluation of ADC-associated PNS safety signals, focusing on signal spectrum, reproducibility, and label–signal concordance. This study was not designed to compare risks between individual ADCs but rather to characterize and externally validate PNS-related disproportionality signals at the preferred-term level to inform clinical awareness and ongoing regulatory surveillance.

## 2 Methods

### 2.1 Data sources and study design

We conducted a multi-database pharmacovigilance study using individual case safety reports (ICSRs) from three national spontaneous reporting systems (SRSs): the U.S. Food and Drug Administration Adverse Event Reporting System (FAERS; 2004Q1–2025 Q3), the Japanese Adverse Drug Event Report (JADER; 2004Q1–2025 Q3) database, and the Canada Vigilance Adverse Reaction Database (CVARD; January 1965 - October 2025) (U.S. Food and Drug Administration, 2025). The study followed a standardized workflow including data harmonization, deduplication, identification of antibody–drug conjugate (ADC) exposure, selection of peripheral nervous system (PNS)–related preferred terms (PTs), disproportionality analysis, and cross-database validation.

Raw quarterly FAERS data were downloaded in ASCII format and imported into R (version 4.5.0). Reports were structured into relational tables (DEMO, DRUG, REAC, OUTC, RPSR, THER, and INDI). Duplicate reports were removed in accordance with FDA recommendations, and records listed in FDA “DELETED” files or containing missing or implausible drug or event information were excluded. Drug names were standardized using Drugs@FDA reference lists, and demographic variables were normalized during preprocessing (U.S. Food and Drug Administration, 2026). Analyses were restricted to reports submitted by healthcare professionals (medical doctors, pharmacists, and other healthcare professionals) to enhance data reliability. Duplicate reports in the JADER and CVARD databases were removed based on the unique case or report identifiers available in each database. To ensure baseline comparability with the FAERS analysis, reports in the JADER and CVARD databases were restricted to those in which the study drug was recorded as the primary suspect (PS) and the reporter was a healthcare professional (MD, PH, or HP).

Adverse events in FAERS, JADER, and CVARD were coded using the Medical Dictionary for Regulatory Activities (MedDRA), version 27.1, with cross-lingual harmonization applied to ensure consistent mapping of preferred terms across databases (MedDRA Maintenance and Support Services Organization (MSSO), 2025).

### 2.2 Identification of ADC exposure and PNS-Related events

All FDA-approved ADCs available through October 2025 were included, covering major payload classes, including DM1, vedotin, mafodotin, deruxtecan, and tesirine. ADC exposure was identified by screening standardized generic, brand, and active substance names in the DRUG tables of each database. Reports were restricted to those in which the ADC was recorded as the primary suspect, consistent with regulatory guidance; analogous role classifications were applied in JADER and CVARD.

PNS-related adverse events were identified using a MedDRA Standardised MedDRA Query (SMQ)–guided approach, with the SMQ “Peripheral neuropathy” serving as the initial framework for case identification (MedDRA Maintenance and Support Services Organization (MSSO), 2025). To ensure comprehensive capture of clinically relevant peripheral nervous system toxicity, the SMQ-derived preferred terms were supplemented through expert clinical review with additional MedDRA preferred terms from the System Organ Class “Nervous system disorders” that were clearly attributable to peripheral rather than central nervous system involvement.

This process yielded a curated set of 121 PNS-related preferred terms encompassing peripheral neuropathy, polyneuropathy, sensory and motor neuropathies, Guillain–Barré syndrome, chronic inflammatory demyelinating polyradiculoneuropathy, radiculopathies, autonomic neuropathies, and selected cranial nerve disorders. Central nervous system disorders were excluded to preserve specificity. The same PNS-related preferred term set was applied consistently across FAERS, JADER, and CVARD.

### 2.3 Disproportionality analysis

Disproportionality analyses were conducted independently within each spontaneous reporting system (FAERS, JADER, and CVARD) using standard 2 × 2 contingency tables constructed at the report level for each antibody–drug conjugate (ADC)–preferred term (PT) pair.

To minimize the impact of duplicate reporting, analyses were performed using unique reports as provided by each database after application of database-specific deduplication procedures (FAERS: CASEID with the most recent FDA_DT retained; JADER and CVARD: database-provided unique report identifiers). Only reports in which the ADC was recorded as the primary suspect drug were included in signal detection analyses.

For each ADC–PT pair, the comparator background consisted of all other reports in the same database involving drugs other than the index ADC and the same PT (case–noncase design). Associations were quantified using four established disproportionality metrics: reporting odds ratio (ROR) with 95% confidence intervals, proportional reporting ratio (PRR) with χ^2^ statistics, information component (IC), and empirical Bayes geometric mean (EBGM) with its lower one-sided 90% credibility interval (EBGM05) (van Puijenbroek et al., 2002; Bate et al., 1998; Szarfman et al., 2002).

A positive signal was defined according to commonly adopted thresholds: (1) a lower bound of the 95% confidence interval of the ROR greater than 1; (2) PRR ≥2 with χ^2^ ≥ 4; (3) the lower bound of the 95% confidence interval of the information component (IC025) > 0; (4) the lower one-sided 90% credibility interval of the empirical Bayesian geometric mean (EBGM05) ≥ 2; and (5) at least three co-occurrence reports for the drug–event pair. Signals were considered positive only when all four disproportionality algorithms met their predefined thresholds. PTs with fewer than three reports were excluded from disproportionality calculations to reduce statistical instability. No adjustments for multiple comparisons were applied, consistent with the exploratory and hypothesis-generating nature of spontaneous reporting system analyses (van Puijenbroek et al., 2002; Bate et al., 1998).

Temporal trends were explored using trend-based ROR analyses aligned with the calendar year of regulatory approval for each ADC to visualize changes in reporting patterns over time. Consistent with the exploratory nature of pharmacovigilance signal detection, no individual case-level causality assessment was performed (Szarfman et al., 2002).

### 2.4 Sensitivity analysis

To evaluate the robustness of the study findings and reduce potential confounding caused by concomitant medications known to be associated with peripheral neuropathy, a sensitivity analysis was conducted using the FAERS database, which served as the primary dataset for signal detection.

In this analysis, reports involving drugs known to cause peripheral neurotoxicity were excluded (Lee et al., 2024), including paclitaxel, docetaxel, nab-paclitaxel, cabazitaxel, cisplatin, carboplatin, oxaliplatin, vincristine, vinblastine, vinorelbine, bortezomib, and thalidomide.

Apart from this exclusion criterion, all other data filtering conditions (e.g., reporter type and restriction to primary suspect drugs) were consistent with those used in the primary analysis.

### 2.5 Cross-database validation and prescribing information review

Signals identified in FAERS were assessed for reproducibility in JADER and CVARD using the same disproportionality thresholds. Validation focused on consistency in effect direction (ROR >1) and confidence intervals not crossing unity in at least two databases. PTs that were absent or too sparse in JADER or CVARD were classified as not evaluable.

Prescribing information approved by the U.S. Food and Drug Administration (FDA) was systematically reviewed for all included ADCs. PNS-related adverse reactions described in FDA labeling were mapped to MedDRA preferred terms (PTs). Post-marketing PNS signals identified in FAERS were then classified as either known PNS adverse events (explicitly listed in FDA prescribing information) or new PNS signals (identified in FAERS but absent from FDA labeling). Concordance between PI content and post-marketing signals was summarized using a stacked bar chart and a PT-by-ADC heatmap.

### 2.6 Descriptive analyses

Descriptive analyses summarized demographic characteristics, reporter type, geographic distribution, and seriousness outcomes, including death, life-threatening events, hospitalization, and disability. Time-to-onset was calculated using therapy start and event dates when available and visualized using empirical cumulative distribution functions and box-and-whisker plots. Annual reporting trends were examined using absolute counts and proportional contributions by ADC.

### 2.7 Statistical software

All data processing and statistical analyses were performed using R (version 4.5.0). Data visualization was performed using Python. Data processing and statistical analyses were performed using standard packages (R: *data.table*, *dplyr*, *epiR*, *vcd*; Python: *pandas*, *numpy*, *matplotlib*, *seaborn*). Figures were generated using *ggplot2*, *matplotlib*, and *seaborn*.

## 3 Results

### 3.1 Descriptive analysis

**FIGURE 1:**
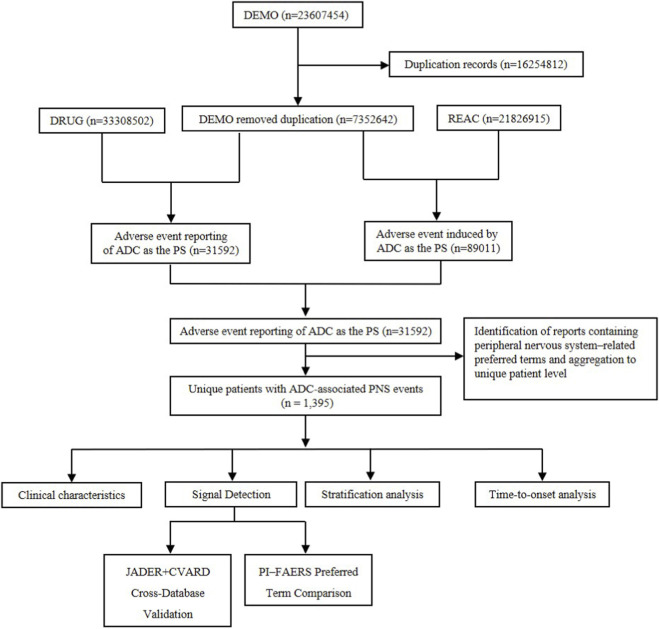
Study workflow for the identification of antibody–drug conjugate–associated peripheral nervous system adverse events. Flow chart illustrating the identification of post-marketing reports of peripheral nervous system (PNS) adverse events associated with antibody–drug conjugates (ADCs) in the FAERS database from 2004 Q1 to 2025 Q3. Reports were restricted to cases in which an ADC was recorded as the primary suspect drug. PNS-related adverse events were identified using the MedDRA Standardised MedDRA Query “Peripheral neuropathy” supplemented by curated preferred terms. Deduplication was applied according to FDA recommendations to derive unique PNS-related case reports included in the final analytic cohort. FAERS, FDA Adverse Event Reporting System; ADC, antibody–drug conjugate; PNS, peripheral nervous system; PS, primary suspect.

Among all adverse event reports submitted to the FAERS database between 2004 Q1 and 2025 Q3, a total of 31,592 reports in which an antibody–drug conjugate (ADC) was recorded as the primary suspect drug were identified from the post-marketing setting. After restricting these reports to peripheral nervous system (PNS)–related adverse events using the predefined MedDRA Standardised MedDRA Query and curated preferred terms, 1,395 unique PNS-related cases, defined as unique case reports after deduplication, were included in the final analytic cohort (Figure 1).

Baseline characteristics of ADC-related PNS case reports in FAERS are summarized in Table 1. Overall, 1,395 PNS-related cases involving 13 FDA-approved ADCs were identified. Marked heterogeneity was observed across ADCs in demographic characteristics, reporter type, and seriousness outcomes. Sex distributions differed substantially by ADC. Male-predominant reporting was observed for Padcev (77.3%), Mylotarg (75.0%), Polivy (53.2%), and Adcetris (46.3%), whereas female-predominant reporting was noted for Kadcyla (79.7%), Enhertu (90.9%), Trodelvy (100%), Tivdak (88.2%), and Elahere (60.5%). A high proportion of sex-unknown reports was present for Blenrep (73.7%) and Elahere (39.5%), indicating variable completeness of FAERS records.

**TABLE 1:** Baseline characteristics of peripheral nervous system (PNS) reports associated with 13 antibody–drug conjugates (ADCs) in the FAERS database.

Characteristic	Mylotarg	Adcetris	Kadcyla	Besponsa	Polivy	Padcev	Enhertu	Trodelvy	Blenrep	Zynlonta	Tivdak	Elahere	Emrelis
	CD33	CD30	HER2	CD22	CD79b	Nectin-4	HER2	TROP2	BCMA	CD19	Tissue factor (TF)	FRα	c-Met
	N = 8	N = 600	N = 118	N = 10	N = 109	N = 335	N = 66	N = 33	N = 19	N = 1	N = 51	N = 43	N = 2
Sex													
Male	6 (75.0%)	278 (46.3%)	2 (1.7%)	2 (20.0%)	58 (53.2%)	259 (77.3%)	4 (6.1%)	0 (0%)	0 (0%)	0 (0%)	2 (3.9%)	0 (0%)	0 (0%)
Female	2 (25.0%)	200 (33.3%)	94 (79.7%)	7 (70.0%)	36 (33.0%)	69 (20.6%)	60 (90.9%)	33 (100%)	5 (26.3%)	1 (100%)	45 (88.2%)	26 (60.5%)	1 (50.0%)
Unknown	0 (0%)	122 (20.3%)	22 (18.6%)	1 (10.0%)	15 (13.8%)	7 (2.1%)	2 (3.0%)	0 (0%)	14 (73.7%)	0 (0%)	4 (7.8%)	17 (39.5%)	1 (50.0%)
Age (year)													
<2	0 (0%)	0 (0%)	0 (0%)	0 (0%)	0 (0%)	0 (0%)	0 (0%)	0 (0%)	0 (0%)	0 (0%)	0 (0%)	0 (0%)	0 (0%)
2∼11	0 (0%)	5 (0.8%)	0 (0%)	1 (10.0%)	0 (0%)	0 (0%)	0 (0%)	0 (0%)	0 (0%)	0 (0%)	0 (0%)	0 (0%)	0 (0%)
12∼17	0 (0%)	10 (1.7%)	0 (0%)	1 (10.0%)	0 (0%)	0 (0%)	0 (0%)	0 (0%)	0 (0%)	0 (0%)	0 (0%)	0 (0%)	0 (0%)
18∼64	4 (50.0%)	245 (40.8%)	50 (42.4%)	1 (10.0%)	20 (18.3%)	62 (18.5%)	24 (36.4%)	19 (57.6%)	2 (10.5%)	1 (100%)	10 (19.6%)	9 (20.9%)	1 (50.0%)
65∼85	4 (50.0%)	114 (19.0%)	13 (11.0%)	5 (50.0%)	51 (46.8%)	190 (56.7%)	9 (13.6%)	4 (12.1%)	1 (5.3%)	0 (0%)	2 (3.9%)	1 (2.3%)	1 (50.0%)
>85	0 (0%)	5 (0.8%)	0 (0%)	0 (0%)	8 (7.3%)	10 (3.0%)	0 (0%)	1 (3.0%)	0 (0%)	0 (0%)	0 (0%)	0 (0%)	0 (0%)
Unknown	0 (0%)	221 (36.8%)	55 (46.6%)	2 (20.0%)	30 (27.5%)	73 (21.8%)	33 (50.0%)	9 (27.3%)	16 (84.2%)	0 (0%)	39 (76.5%)	33 (76.7%)	0 (0%)
Reporter													
Health professional	0 (0%)	103 (17.2%)	20 (16.9%)	2 (20.0%)	8 (7.3%)	96 (28.7%)	28 (42.4%)	9 (27.3%)	1 (5.3%)	0 (0%)	21 (41.2%)	15 (34.9%)	0 (0%)
Medical doctor	8 (100%)	460 (76.7%)	83 (70.3%)	7 (70.0%)	95 (87.2%)	209 (62.4%)	34 (51.5%)	21 (63.6%)	18 (94.7%)	1 (100%)	25 (49.0%)	23 (53.5%)	2 (100%)
Pharmacist	0 (0%)	37 (6.2%)	15 (12.7%)	1 (10.0%)	6 (5.5%)	30 (9.0%)	4 (6.1%)	3 (9.1%)	0 (0%)	0 (0%)	5 (9.8%)	5 (11.6%)	0 (0%)
Outcome													
Death	0 (0%)	28 (4.7%)	2 (1.7%)	0 (0%)	4 (3.7%)	24 (7.2%)	5 (7.6%)	5 (15.2%)	5 (26.3%)	0 (0%)	0 (0%)	0 (0%)	0 (0%)
Life-threatening	0 (0%)	9 (1.5%)	0 (0%)	2 (20.0%)	0 (0%)	0 (0%)	0 (0%)	0 (0%)	0 (0%)	0 (0%)	0 (0%)	0 (0%)	0 (0%)
Hospitalization	6 (75.0%)	114 (19.0%)	16 (13.6%)	1 (10.0%)	28 (25.7%)	54 (16.1%)	9 (13.6%)	4 (12.1%)	2 (10.5%)	1 (100%)	4 (7.8%)	0 (0%)	1 (50.0%)
Disability	2 (25.0%)	34 (5.7%)	7 (5.9%)	2 (20.0%)	2 (1.8%)	10 (3.0%)	1 (1.5%)	0 (0%)	0 (0%)	0 (0%)	3 (5.9%)	1 (2.3%)	0 (0%)
Congenital anomaly	0 (0%)	0 (0%)	0 (0%)	0 (0%)	0 (0%)	0 (0%)	0 (0%)	0 (0%)	0 (0%)	0 (0%)	0 (0%)	0 (0%)	0 (0%)
Required intervention	0 (0%)	2 (0.3%)	0 (0%)	0 (0%)	0 (0%)	0 (0%)	0 (0%)	0 (0%)	0 (0%)	0 (0%)	0 (0%)	0 (0%)	0 (0%)
Other serious	0 (0%)	367 (61.2%)	61 (51.7%)	5 (50.0%)	64 (58.7%)	241 (71.9%)	38 (57.6%)	24 (72.7%)	12 (63.2%)	0 (0%)	42 (82.4%)	27 (62.8%)	0 (0%)
Unknown	0 (0%)	46 (7.7%)	32 (27.1%)	0 (0%)	11 (10.1%)	6 (1.8%)	13 (19.7%)	0 (0%)	0 (0%)	0 (0%)	2 (3.9%)	15 (34.9%)	1 (50.0%)

Data are presented as case counts with percentages. The table summarizes demographic characteristics, age distribution, reporter types, and clinical seriousness outcomes for peripheral nervous system (PNS) adverse event reports attributed to each ADC.

Adults comprised the majority of PNS-related cases. The 18–64-year age group accounted for a large share of reports for several ADCs (e.g., Adcetris 40.8%, Kadcyla 42.4%, Trodelvy 57.6%), whereas older adults aged 65–85 years contributed prominently for Padcev (56.7%) and Polivy (46.8%). Pediatric reports were infrequent across agents. Notably, age information was frequently missing for several ADCs, particularly Blenrep (84.2%), Elahere (76.7%), and Adcetris (36.8%).

Medical doctors accounted for the largest proportion of reports across most ADCs (e.g., Polivy 87.2%, Padcev 62.4%, Trodelvy 63.6%, Blenrep 94.7%), whereas pharmacist-submitted reports comprised a smaller fraction (e.g., Kadcyla 12.7%, Adcetris 6.2%). Serious outcomes were commonly reported. Hospitalization was frequently recorded across multiple ADCs (e.g., Adcetris 19.0%, Polivy 25.7%, Padcev 16.1%). Death was reported in several ADC groups, including Adcetris (4.7%) and Padcev (7.2%), and a higher proportion was observed for Blenrep (26.3%); however, for some ADCs the underlying denominators were small and interpretation should consider report volume.

**FIGURE 2:**
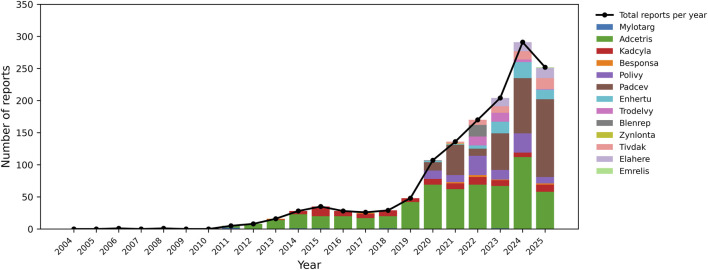
Annual distribution of antibody–drug conjugate (ADC)-related peripheral neuropathy reports in FAERS (2004–2025). Annual reporting trends of PNS adverse events associated with 13 FDA-approved ADCs in FAERS from 2004 to 2025. PNS events were defined using a predefined set of 121 MedDRA preferred terms identified through a Standardised MedDRA Query–guided and expert-curated approach. Stacked bars represent yearly PNS-related case reports by ADC, and the black line indicates the total annual number of ADC-associated PNS case reports.

Across 2004–2025, annual reporting of ADC-associated PNS cases in FAERS remained low for more than a decade following the initial ADC approvals, with fewer than 20 PNS-related cases reported per year before 2015. Reporting increased gradually from 2016 onward and accelerated markedly after 2020, coinciding with the availability and broader clinical uptake of multiple newer ADCs. Total annual PNS-related cases increased from 55 in 2019 to more than 150 by 2022, peaking at nearly 300 in 2024, followed by a modest decline in 2025 (Figure 2). Adcetris contributed the largest share of PNS-related cases, while the cumulative contribution of newer ADCs (e.g., Padcev, Polivy, Enhertu, Trodelvy, and Blenrep) increased after their market entry, shifting the overall composition of annual reporting. These patterns indicate a substantial recent rise in reported ADC-associated PNS events in FAERS and underscore the relevance of continued post-marketing surveillance as ADC use expands.

**FIGURE 3:**
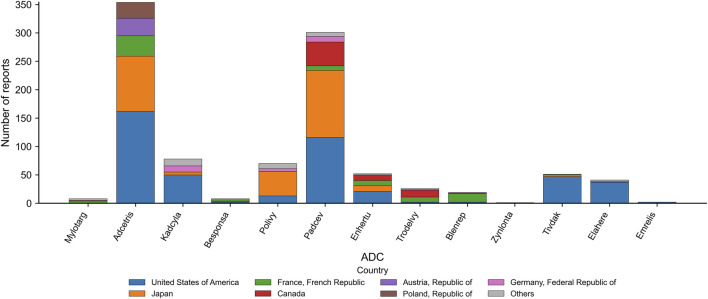
Country distribution of ADC-related peripheral neuropathy reports. Stacked bars depict the number of peripheral neuropathy reports attributed to each ADC, stratified by reporting country. The United States accounted for the largest proportion of submissions, followed by Japan, France, Canada, Austria, Poland, Germany, and the United Kingdom; all remaining countries were grouped as “Others”. This distribution reflects differences in regional market availability and uptake of individual ADCs.

Geographical reporting patterns showed clear variation across ADCs (Figure 3). For most products—including Adcetris, Padcev, and Polivy—the United States contributed the majority of peripheral neuropathy cases, reflecting early approval and high clinical uptake. Adcetris demonstrated the broadest international distribution, with additional reports from Japan, Europe, and Canada. In contrast, newer ADCs such as Trodelvy, Elahere, and Emrelis showed more limited geographic spread, with cases reported predominantly from the United States. Overall, reporting volume closely aligned with regional market availability and duration of commercial use.

### 3.2 Disproportionality analysis

**FIGURE 4:**
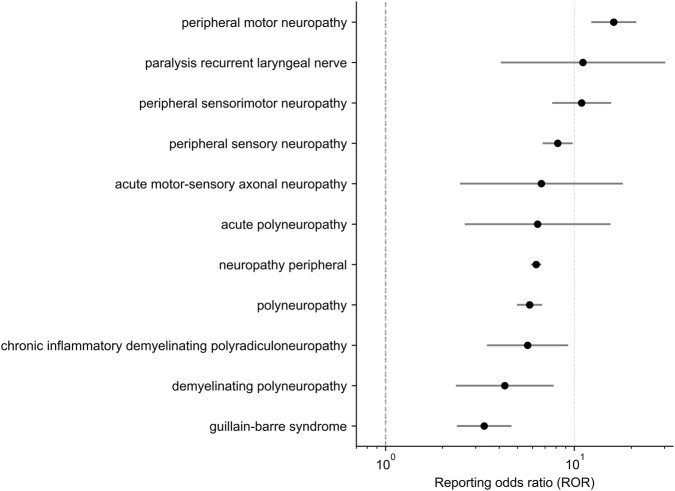
Forest plot of peripheral nervous system (PNS)–related safety signals associated with antibody–drug conjugates (ADCs) identified in the FAERS database. The plot displays reporting odds ratios (RORs) with 95% confidence intervals (CIs) for selected PNS preferred terms (PTs). Point estimates represent ROR values, and horizontal lines indicate corresponding 95% CIs. The vertical dashed line denotes the null value (ROR = 1). PTs are ordered by increasing ROR to facilitate comparison of signal strength. An x-axis logarithmic scale is used to improve visualization across a wide range of effect sizes.

Disproportionality analysis in FAERS identified multiple peripheral nervous system (PNS)–related preferred terms (PTs) with statistically significant associations with antibody–drug conjugates (Figure 4). For all PTs shown, reporting odds ratios (RORs) exceeded unity, and the lower bounds of the 95% confidence intervals were greater than 1, meeting the predefined criteria for positive disproportionality signals.

Among the strongest signals, peripheral motor neuropathy and peripheral sensorimotor neuropathy showed elevated ROR estimates with relatively narrow confidence intervals, indicating consistent disproportionate reporting in FAERS.

In addition to these common phenotypes, several less frequently reported but clinically severe neuropathic conditions were also identified as positive signals, including acute motor–sensory axonal neuropathy, acute polyneuropathy, chronic inflammatory demyelinating polyradiculoneuropathy, and Guillain–Barré syndrome. These PTs were characterized by wider confidence intervals, consistent with lower case counts in spontaneous reporting systems, but nevertheless satisfied all signal detection thresholds. Other PNS-related PTs exhibited heterogeneous signal strengths and are detailed in Figure 4.

Overall, the forest plot illustrates a broad spectrum of ADC-associated PNS safety signals in FAERS, encompassing common sensory and motor neuropathies as well as rarer demyelinating and immune-mediated syndromes. The coexistence of high-frequency, high-precision signals and lower-frequency but clinically serious PTs underscores the heterogeneity of post-marketing neurotoxicity patterns associated with ADC therapy.

### 3.3 Sensitivity analysis

A sensitivity analysis was conducted after excluding reports involving drugs known to cause peripheral neuropathy, eight peripheral nervous system–related preferred terms remained significant in the disproportionality analysis: peripheral motor neuropathy, peripheral sensorimotor neuropathy, peripheral sensory neuropathy, peripheral neuropathy, polyneuropathy, demyelinating polyneuropathy, acute polyneuropathy, and Guillain–Barré syndrome (Supplementary Figure S1; Supplementary Table S1).

Compared with the primary analysis, signals for acute motor-sensory axonal neuropathy, chronic inflammatory demyelinating polyradiculoneuropathy, and recurrent laryngeal nerve paralysis were not detected in the sensitivity analysis.

### 3.4 Cross-database disproportionality analysis

Across the three spontaneous reporting systems, peripheral neuropathy showed a consistent and statistically significant disproportionality signal associated with antibody–drug conjugates (ADCs) (Table 2).

**TABLE 2:** Overall validation of peripheral nervous system (PNS)–related safety signals across three national spontaneous reporting systems.

Database	a	b	c	d	ROR	95% CI	Signal status*
FAERS	1,477	87,534	79,020	21,658,884	4.62	4.39–4.87	Positive
JADER	836	6,018	3,470	1,070,218	42.84	39.56–46.40	Positive
CVARD	219	11,046	3,927	4,117,708	20.79	18.12–23.85	Positive

Abbreviations: FAERS, FDA Adverse Event Reporting System; JADER, Japanese Adverse Drug Event Report database; CVARD, Canada Vigilance Adverse Reaction Database; ROR, reporting odds ratio; CI, confidence interval.

a denotes ADC–peripheral neuropathy co-occurrences at the preferred-term (PT) level meeting predefined signal detection criteria, with a single report contributing to multiple PTs; b, reports with ADC exposure and other adverse events; c, reports with peripheral neuropathy associated with other drugs; d, reports with other drugs and other adverse events. A positive disproportionality signal was defined as a lower bound of the 95% CI of the ROR >1.

*denotes a positive finding/result.

In FAERS, 1,477 ADC–peripheral neuropathy co-occurrences were identified, yielding an ROR of 4.62 (95% CI 4.39–4.87). This signal met all predefined criteria across the four disproportionality methods (ROR, PRR, IC, and EBGM). Consistent positive signals were also observed in JADER (836 co-occurrences; ROR 42.84, 95% CI 39.56–46.40) and CVARD (219 co-occurrences; ROR 20.79, 95% CI 18.12–23.85).

Across all three databases, ROR estimates were elevated with confidence intervals entirely above unity, indicating directionally concordant and statistically robust signals. This cross-database consistency supports a robust and reproducible signal and reduces the likelihood of database-specific artifacts.

### 3.5 Cross-database validation of PNS-related signals

**FIGURE 5:**
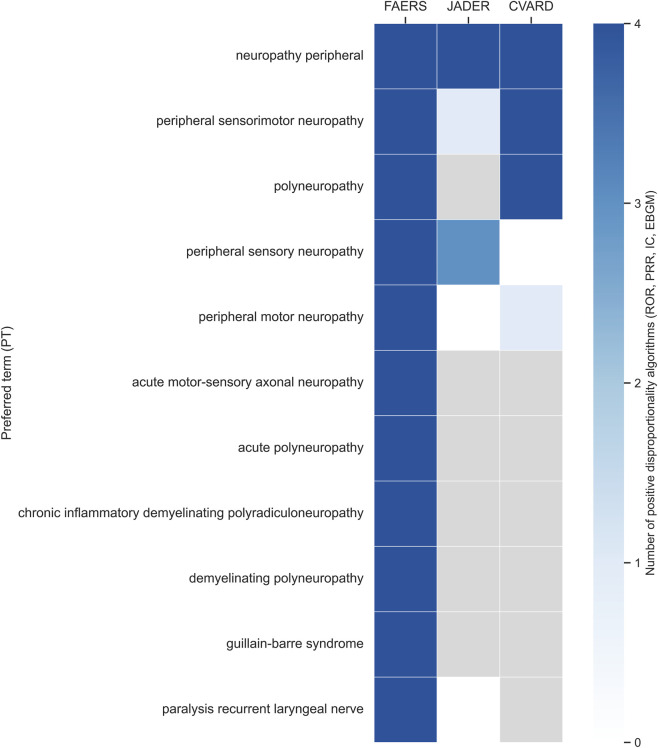
Cross-database validation of peripheral nervous system–related preferred terms. Heatmap summarizing the number of disproportionality algorithms supporting each peripheral nervous system–related preferred term across FAERS, JADER, and CVARD. Values range from 0 to 4, representing the number of algorithms identifying a positive signal within each database. Darker blue shading indicates support by a greater number of algorithms, white cells indicate no positive signal, and Gy cells denote preferred terms not evaluable due to insufficient reports. Preferred terms are ordered by cross-database replication strength.

Cross-database validation demonstrated a high degree of reproducibility for major peripheral nervous system (PNS)–related preferred terms (PTs) identified in FAERS (Table 3; Figure 5). Peripheral neuropathy showed the most robust and consistent signal, with concordant positivity across all four disproportionality algorithms in FAERS, JADER, and CVARD, indicating a reproducible association across three independent spontaneous reporting systems. Peripheral sensorimotor neuropathy likewise exhibited strong cross-database support, with multiple positive algorithms confirmed in all three databases.

**TABLE 3:** Cross-database validation of peripheral neuropathy–related Preferred Terms across FAERS, JADER, and CVARD.

PT (Preferred term)	FAERS	JADER	CVARD
Neuropathy peripheral	4	4	4
Polyneuropathy	4	NA	4
Peripheral sensory neuropathy	4	3	0
Peripheral motor neuropathy	4	0	1
Guillain-barre syndrome	4	NA	NA
Peripheral sensorimotor neuropathy	4	3	4
Chronic inflammatory demyelinating polyradiculoneuropathy	4	NA	NA
Demyelinating polyneuropathy	4	NA	NA
Acute polyneuropathy	4	NA	NA
Acute motor-sensory axonal neuropathy	4	NA	NA
Paralysis recurrent laryngeal nerve	4	0	NA

Abbreviations: FAERS, FDA Adverse Event Reporting System; JADER, Japanese Adverse Drug Event Report database; CVARD, Canada Vigilance Adverse Reaction Database; PT, preferred term; ROR, reporting odds ratio; PRR, proportional reporting ratio; EBGM, empirical Bayes geometric mean; IC, information component.

Values indicate the number of disproportionality algorithms identifying a positive signal for each PT within a given database (maximum = 4). NA indicates that the PT was not reported or could not be evaluated due to insufficient case counts. Signal criteria were predefined (ROR lower 95% CI > 1; PRR ≥2 with χ^2^ ≥ 4; EBGM_05_ > 2; IC_025_ > 0).

Several PTs, including polyneuropathy and peripheral motor neuropathy, demonstrated reproducible signals in FAERS and CVARD but were not confirmed in JADER, whereas peripheral sensory neuropathy showed partial replication, with positive signals in FAERS and JADER but not in CVARD. These discrepancies likely reflect differences in reporting volume and database size rather than inconsistent associations.

In contrast, rarer and more clinically specific PTs—such as Guillain–Barré syndrome, chronic inflammatory demyelinating polyradiculoneuropathy, demyelinating polyneuropathy, acute polyneuropathy, and acute motor-sensory axonal neuropathy—were strongly supported in FAERS but were absent or not evaluable in JADER and CVARD due to limited case counts. Overall, common neuropathy phenotypes demonstrated the highest cross-database reproducibility, whereas rare PNS events were primarily detectable in FAERS, highlighting both the robustness of the major signals and the sensitivity limitations of smaller spontaneous reporting systems.

### 3.6 Time to onset

**FIGURE 6:**
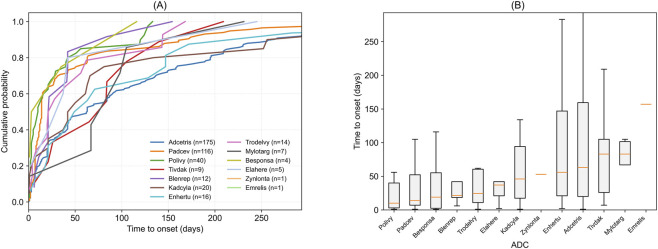
Time to onset of peripheral neuropathy associated with antibody–drug conjugates (ADCs). **(A)** Empirical cumulative distribution function (ECDF) curves based on evaluable reports with available therapy start and event dates; numbers of cases for each ADC are shown in the legend. **(B)** Box-and-whisker plots summarizing onset-time distributions, with medians and interquartile ranges displayed. ADCs with limited case numbers are included for completeness and should be interpreted with caution.

Time-to-onset analysis revealed substantial heterogeneity in the temporal patterns of ADC-associated peripheral neuropathy (Figure 6). Several ADCs, including Polivy, Padcev, Blenrep, Elahere, and Trodelvy, were associated with relatively early onset, with more than half of reported events occurring within approximately 20–40 days after treatment initiation. In contrast, Adcetris, Enhertu, and Kadcyla showed more gradual accumulation of events, with a considerable proportion occurring beyond 80–100 days.

These patterns were consistent across ECDF curves and box-and-whisker plots, which further demonstrated longer and more variable onset distributions for Adcetris and Enhertu, including late-onset events exceeding 200 days. Tivdak exhibited an intermediate onset profile with substantial variability. Overall, ADCs displayed distinct onset patterns ranging from early-onset to delayed and heterogeneous presentations.

### 3.7 Severity of ADC-associated peripheral neuropathy

**FIGURE 7:**
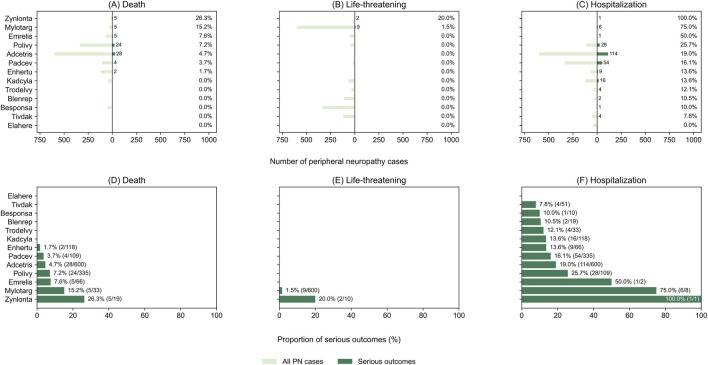
Severity of antibody–drug conjugate (ADC)–associated peripheral neuropathy in FAERS. Panels **(A–C)** show the distribution of serious outcomes among ADC-associated peripheral neuropathy cases, including death **(A)**, life-threatening events **(B)**, and hospitalization **(C)**, presented as absolute numbers. Mirrored horizontal bar charts depict the total number of peripheral neuropathy cases for each ADC (left) and the corresponding number with serious outcomes (right). Panels **(D–F)** display the proportions of the same outcomes, expressed as percentages and ordered by outcome-specific severity rates, with percentages calculated using the total number of peripheral neuropathy cases for each ADC as the denominator. FAERS, Food and Drug Administration Adverse Event Reporting System; ADC, antibody–drug conjugate.

As shown in Figures 7A–C, the absolute numbers of death, life-threatening events, and hospitalization among ADC-associated peripheral neuropathy cases varied across products and generally tracked the overall volume of peripheral neuropathy reports for each ADC.

In the proportional analysis (Figures 7D–F), heterogeneity in severity rates was observed across ADCs: hospitalization accounted for the largest share of serious outcomes for several products, whereas deaths were comparatively infrequent across most ADCs.

For ADCs with small numbers of peripheral neuropathy reports, the proportional severity could appear high, highlighting the influence of limited denominators in spontaneous reporting data.

### 3.8 PI vs. post-marketing PNS signals

**FIGURE 8:**
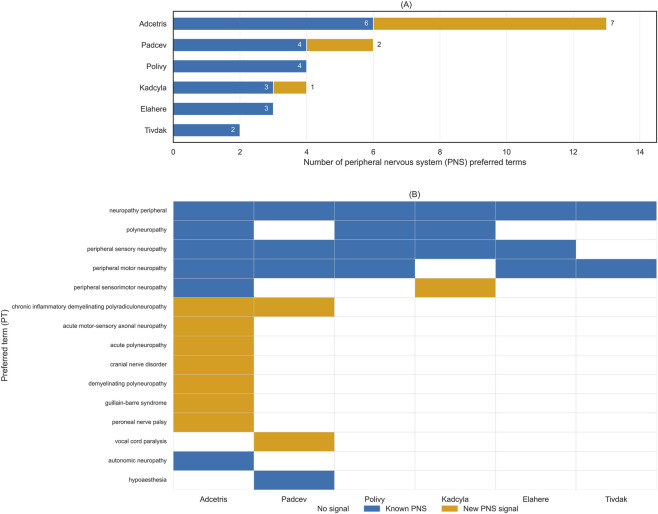
Known and newly identified peripheral nervous system (PNS) adverse event signals across antibody–drug conjugates. **(A)** Number of PNS preferred terms (PTs) per ADC, categorized as known events listed in prescribing information or newly identified FAERS signals. **(B)** PT-level distribution of known PNS events and FAERS-positive signals across ADCs. Newly identified signals were defined as PTs positive by all four disproportionality algorithms in FAERS. PTs were ordered with commonly reported neuropathy phenotypes shown first, followed by newly identified signals prioritized by the number of ADCs involved.

Across antibody–drug conjugates, both established and newly identified peripheral nervous system (PNS) safety signals were observed (Figure 8). The number of PNS preferred terms (PTs) varied substantially across ADCs, indicating heterogeneity in the scope of reported PNS involvement (Figure 8A).

At the PT level, common neuropathy phenotypes—such as peripheral neuropathy, polyneuropathy, and peripheral sensory or motor neuropathy—were consistently observed across multiple ADCs and were largely concordant with events listed in prescribing information (Figure 8B). In contrast, several less frequent and more clinically specific PNS PTs, including demyelinating, acute, or focal neuropathies, emerged as FAERS-positive signals in selected ADCs and were not uniformly distributed across agents, reflecting agent-specific reporting patterns.

Overall, these findings indicate a shared core of well-recognized PNS adverse events alongside ADC-specific patterns of newly identified PNS signals not consistently captured in current labeling, underscoring heterogeneity in the neurotoxicity profiles of different ADCs.

## 4 Discussion

### 4.1 Overview of key safety signals

In this multi-database pharmacovigilance study integrating FAERS, JADER, and CVARD, we identified consistent and clinically meaningful safety signals for peripheral nervous system (PNS) adverse events associated with antibody–drug conjugates (ADCs) (Fusaroli et al., 2024a; Fusaroli et al., 2024b). Across three independent spontaneous reporting systems, several core PNS preferred terms—most notably peripheral neuropathy, polyneuropathy, and peripheral sensorimotor neuropathy—demonstrated robust and reproducible disproportionality patterns, supporting a stable association at the signal-detection level (Fusaroli et al., 2024a; Fusaroli et al., 2024b; Wiśniewski et al., 2016; European Medicines Agency, 2017).

These findings extend prior FAERS-based analyses by demonstrating cross-database reproducibility and by characterizing the broader spectrum of PNS phenotypes beyond aggregate neuropathy categories (Yamaoka et al., 2025; Chen et al., 2024). The observation that major PNS signals were directionally concordant across databases with distinct reporting environments strengthens confidence that these associations are unlikely to be attributable to database-specific artifacts alone and instead reflect a genuine post-marketing safety signal (Fusaroli et al., 2024a; Fusaroli et al., 2024b; Wiśniewski et al., 2016).

Importantly, comparison with FDA prescribing information revealed that several PNS preferred terms identified in post-marketing surveillance were not consistently captured in current labeling. This gap highlights the potential divergence between pre-approval safety characterization and real-world clinical experience and underscores the importance of ongoing pharmacovigilance as ADC use expands across indications and treatment lines (Croteau et al., 2022; Insani et al., 2018).

### 4.2 Clinical patterns of ADC-associated peripheral neuropathy

From a clinical and pharmacological perspective, the observed heterogeneity in time to onset of ADC-associated peripheral neuropathy likely reflects differences in payload mechanisms, intracellular processing, and cumulative neurotoxic potential rather than a uniform class effect (Best et al., 2021; Fu et al., 2023; Starobova and Vetter, 2017; D’Souza et al., 2025; Loprinzi et al., 2020). Vedotin-based ADCs tended to exhibit earlier-onset neuropathy, consistent with the acute axonal injury induced by microtubule disruption, whereas ADCs carrying non–microtubule-disrupting payloads showed more delayed or heterogeneous onset patterns, plausibly related to cumulative cellular stress or indirect neuronal injury (Best et al., 2021; Fu et al., 2023; Starobova and Vetter, 2017). These temporal patterns should therefore be interpreted as supportive contextual evidence for mechanistic heterogeneity rather than as indicators of comparative risk or causality (Fusaroli et al., 2024a; Wiśniewski et al., 2016).

In addition to payload-related mechanisms, structural characteristics of ADCs may further contribute to this heterogeneity. Linker stability is a key determinant of the timing and location of payload release, where insufficient stability may result in premature systemic exposure, while more stable linkers require intracellular processing and may be associated with delayed toxicity onset (Tsuchikama and An, 2018; Su et al., 2021). Moreover, cleavable linkers have been associated with distinct toxicity profiles, including a higher propensity for peripheral neuropathy, likely due to increased release of membrane-permeable payloads (Donaghy, 2016; Nguyen et al., 2023). In parallel, the bystander effect may also contribute to off-target neurotoxicity, as membrane-permeable payloads can diffuse from target cells into adjacent tissues, thereby extending cytotoxic effects beyond antigen-positive cells (Ogitani et al., 2016b). These mechanisms provide a biologically plausible explanation for the variability in TTO observed across different ADCs.

Serious clinical outcomes were reported across multiple ADCs. In descriptive analyses, higher proportions of hospitalization and life-threatening neuropathic events were observed for certain agents, highlighting the clinical relevance of early recognition and timely management in routine practice (Starobova and Vetter, 2017; D’Souza et al., 2025; Loprinzi et al., 2020). Although deaths were uncommon, their occurrence across different ADC categories suggests that peripheral neuropathy may, in some cases, reflect broader treatment-related toxicity or contribute indirectly to clinically meaningful treatment interruptions (Starobova and Vetter, 2017; Loprinzi et al., 2020).

Overall, these findings indicate substantial heterogeneity in both onset and severity profiles of ADC-associated neuropathy, emphasizing that monitoring strategies may need to be adapted to specific ADCs and payload classes rather than applied uniformly across the class (Starobova and Vetter, 2017; D’Souza et al., 2025; Loprinzi et al., 2020).

Clinical trial evidence further supports the occurrence of peripheral neuropathy associated with antibody–drug conjugates (ADCs). In the phase III ECHELON-1 randomized controlled trial (Connors et al., 2018; Straus et al., 2021), peripheral neuropathy was reported in 67% (442/662) of patients receiving brentuximab vedotin plus AVD (A + AVD), compared with 43% (286/659) in the ABVD group, with grade ≥3 events occurring in 11% and 2% of patients, respectively. Further safety analyses indicated that sensory neuropathy was the predominant subtype, with peripheral sensory neuropathy occurring in 29% (189/662) of patients in the A + AVD group versus 17% (111/659) in the ABVD group; grade ≥3 events were reported in 5% and <1% of patients, respectively. Notably, long-term follow-up data showed that although most patients experienced resolution or improvement of neuropathy (85.6%), a substantial proportion had persistent peripheral neuropathy (18.9% in the A + AVD group), including ongoing grade ≥2 events in 5.7% of patients (Ansell et al., 2022). These findings suggest that this toxicity is not only common but may also have long-term functional consequences, underscoring its clinical relevance.

Similar findings were observed in the phase III EV-301 trial (Rosenberg et al., 2023), in which peripheral neuropathy was reported in 48.0% of patients treated with enfortumab vedotin compared with 31.6% in the chemotherapy group, with grade ≥3 events occurring in 7.4% and 2.7% of patients, respectively. Sensory neuropathy was the predominant subtype, with an incidence of 45.6% in the enfortumab vedotin group, whereas motor neuropathy was less common (7.8%).

Furthermore, in a phase II study of another ADC, disitamab vedotin, peripheral sensory neuropathy was also among the most frequently reported adverse events, with an incidence of 68.2% and grade ≥3 events in 18.7% of patients (Sheng et al., 2024), further supporting a class effect of ADC-related neurotoxicity.

Collectively, these findings indicate that peripheral neuropathy, particularly sensory neuropathy, represents a clinically meaningful toxicity associated with ADC therapy. The peripheral nervous system adverse event signals identified in our pharmacovigilance analysis are consistent with these clinical trial findings, further supporting a potential association between ADC therapy and neurotoxicity.

### 4.3 Cross-database reproducibility and labeling gaps

A major strength of this study lies in the validation of safety signals across three independent national spontaneous reporting systems. Several core PNS preferred terms—particularly peripheral neuropathy, polyneuropathy, and peripheral sensorimotor neuropathy—were consistently supported across FAERS, JADER, and CVARD using multiple disproportionality algorithms (Fusaroli et al., 2024a; Fusaroli et al., 2024b; Wiśniewski et al., 2016). This cross-database concordance strengthens confidence in these signals beyond a single reporting environment and reduces the likelihood that the findings reflect database-specific artifacts (Fusaroli et al., 2024a; Fusaroli et al., 2024b; Wiśniewski et al., 2016; European Medicines Agency, 2017).

In contrast, several rarer or more clinically specific PNS preferred terms, including Guillain–Barré syndrome, chronic inflammatory demyelinating polyneuropathy, and demyelinating polyneuropathy, demonstrated strong signals in FAERS but could not be reliably evaluated in JADER or CVARD due to limited case counts. Such discrepancies are most plausibly attributable to limited statistical power in smaller databases rather than absence of association, and should therefore be interpreted as hypothesis-generating rather than confirmatory (Fusaroli et al., 2024a; Fusaroli et al., 2024b; Wiśniewski et al., 2016).

Importantly, comparison with regulatory prescribing information revealed that multiple PNS events consistently identified in post-marketing data were not included in the labeling of several ADCs. This divergence underscores the complementary role of pharmacovigilance data to pre-approval trials and highlights the need for ongoing signal evaluation to inform regulatory awareness and potential future labeling considerations (Croteau et al., 2022; Insani et al., 2018).

### 4.4 Biological plausibility and mechanistic considerations

The observed spectrum of ADC-associated peripheral neuropathy is biologically plausible and aligns with established mechanisms of payload-related neurotoxicity. Vedotin-based ADCs carrying monomethyl auristatin E (MMAE) disrupt microtubule dynamics and axonal transport, a mechanism that has been associated with dose-dependent sensory and sensorimotor neuropathy (Best et al., 2021; Fu et al., 2023; Starobova and Vetter, 2017). Mafodotin-based payloads may induce similar or, in some settings, more pronounced neurotoxicity due to increased intracellular retention (Best et al., 2021; Ogitani et al., 2016b).

For ADCs incorporating topoisomerase I inhibitor payloads, such as deruxtecan or govitecan, indirect mechanisms—including mitochondrial stress, inflammatory signaling, or cumulative neuronal injury—may contribute to intermediate or delayed-onset neuropathy (D’Souza et al., 2025; Loprinzi et al., 2020; Modi et al., 2020). In addition, rare immune-mediated neuropathic phenotypes, including CIDP and Guillain–Barré syndrome, observed predominantly in FAERS, may reflect immune activation related to the monoclonal antibody component or secondary immune responses triggered by payload-induced cell death (Starobova and Vetter, 2017; Loprinzi et al., 2020).

Beyond payload-specific mechanisms, structural features of ADCs may further shape neurotoxicity profiles. Linker stability governs the timing and location of payload release, with premature systemic release potentially leading to earlier toxicity, whereas more stable linkers requiring intracellular processing may be associated with delayed or heterogeneous onset patterns (Tsuchikama and An, 2018; Su et al., 2021). In addition, the bystander effect, mediated by membrane-permeable payloads, may enable diffusion into adjacent non-target cells, thereby contributing to off-target neurotoxicity (Ogitani et al., 2016b). Together, these factors, in conjunction with payload-related mechanisms, provide a plausible mechanistic basis for the variability in TTO and neurotoxicity observed across ADCs.

Taken together, these findings highlight the mechanistic heterogeneity of ADC-associated neurotoxicity and support the evaluation of PNS risk at the level of both the ADC construct and its payload, rather than considering ADCs as a uniform pharmacological class (Best et al., 2021; Fu et al., 2023; Starobova and Vetter, 2017; D’Souza et al., 2025; Loprinzi et al., 2020).

### 4.5 Regulatory implications, strengths, and limitations

From a regulatory perspective, these findings illustrate the value of multi-database pharmacovigilance for identifying emerging neurotoxicity signals that may not be fully characterized in pre-approval clinical trials or existing prescribing information (Fusaroli et al., 2024a; Fusaroli et al., 2024b; Croteau et al., 2022). The identification of multiple PNS preferred terms absent from current labeling suggests that periodic reassessment of ADC safety profiles may be warranted, particularly as these agents are increasingly used in earlier treatment lines and broader patient populations (Croteau et al., 2022; Insani et al., 2018).

This study benefits from several strengths, including a large aggregated sample size, harmonized analyses across three international spontaneous reporting systems, standardized MedDRA mapping, and the application of multiple disproportionality algorithms to enhance signal robustness (Fusaroli et al., 2024a; Fusaroli et al., 2024b; Wiśniewski et al., 2016). To reduce potential confounding from concomitant medications known to cause peripheral neuropathy, a sensitivity analysis was conducted after excluding reports involving these drugs. Most signals identified in the primary analysis remained detectable after exclusion, consistent with the main analysis and supporting the robustness of the findings. Several phenotypes with relatively low reporting frequencies were no longer observed, which may be related to the limited number of reports for these events. Overall, these results suggest that the core peripheral neuropathy signals associated with ADC therapy are unlikely to be fully explained by concomitant neurotoxic medications.

The integration of prescribing information comparison further enhances the regulatory relevance of the findings (Croteau et al., 2022).

Nevertheless, the inherent limitations of spontaneous reporting systems must be acknowledged. These include underreporting, reporting bias, lack of exposure denominators, variable data completeness, and the inability to establish causality (Wiśniewski et al., 2016; Lopez-Gonzalez et al., 2009; Alatawi and Hansen, 2017). Regional differences in reporting practices may also contribute to inter-database variability (Wiśniewski et al., 2016; Alatawi and Hansen, 2017). Additionally, rare events could not be systematically evaluated in smaller databases, limiting certain cross-database comparisons (Fusaroli et al., 2024a; Fusaroli et al., 2024b; Wiśniewski et al., 2016).

### 4.6 Future directions

Future studies should aim to further characterize the clinical relevance of rare neuropathic phenotypes, explore potential dose–response relationships, and identify patient-level risk factors for ADC-associated neurotoxicity (Starobova and Vetter, 2017; D’Souza et al., 2025; Loprinzi et al., 2020). Integration of real-world clinical datasets, prospective registries, and mechanistic studies may help delineate susceptible subpopulations and inform targeted risk mitigation strategies (Garrison et al., 2007; Schneeweiss, 2014; Eichler et al., 2021). Continued collaboration among regulatory agencies, manufacturers, and clinicians will be essential to refine neuropathy monitoring approaches, guide individualized dose modifications, and support evidence-informed updates to ADC safety labeling (Fusaroli et al., 2024a; Croteau et al., 2022).

## 5 Conclusion

In this multi-database pharmacovigilance study, consistent and reproducible disproportionality signals for peripheral nervous system (PNS) adverse events associated with antibody–drug conjugates were identified across three independent spontaneous reporting systems. Core neuropathy phenotypes, particularly peripheral neuropathy and peripheral sensorimotor neuropathy, showed concordant signals across databases, whereas rarer PNS phenotypes were primarily detected in FAERS and warrant further investigation.

The heterogeneity observed in time-to-onset patterns and seriousness outcomes highlights the clinical relevance of sustained neurological monitoring during ADC therapy. Notably, several PNS signals identified through post-marketing surveillance were not consistently reflected in current FDA prescribing information, underscoring the complementary role of real-world pharmacovigilance in characterizing evolving safety profiles.

Although causality cannot be inferred, these findings provide hypothesis-generating evidence to support clinical awareness, regulatory surveillance, and future mechanistic and epidemiological studies as ADC use continues to expand.

## Data Availability

Publicly available datasets were analyzed in this study. This data can be found here: FAERS: https://www.fda.gov/drugs/questions-and-answers-fdas-adverse-event-reporting-system-faers/fda-adverse-event-reporting-system-faers-public-dashboard (FDA FAERS Public Dashboard) and quarterly ASCII files: https://www.fda.gov/drugs/fda-adverse-event-reporting-system-faers/fda-adverse-event-reporting-system-faers-latest-quarterly-data-files. JADER: https://www.pmda.go.jp/english/safety/reports.html (PMDA Japanese Adverse Drug Event Report database). CVARD: https://www.canada.ca/en/health-canada/services/drugs-health-products/medeffect-canada/adverse-reaction-database.html (Canada Vigilance Adverse Reaction Database).
