# Supplementary material for: Peripheral nervous system safety signals of antibody–drug conjugates: cross-database reproducibility and labeling gaps identified using FAERS, JADER, and CVARD
Source: Front Pharmacol. 2026 Apr 21;17:1786431. doi: 10.3389/fphar.2026.1786431 (PMC13139113; doi:10.3389/fphar.2026.1786431)
Supplement: Supplementary file 1 [file DataSheet1.pdf]

Supplemental Materials Content

Supplementary Table S1. Disproportionality results for peripheral nervous system (PNS) preferred terms in the sensitivity analysis after excluding reports involving neurotoxic co-medications.

PT	a	ROR(95%Cl)	PRR(χ2))	EBGM(EBGM05)	IC(IC025)
neuropathy peripheral	782	5.79 (5.4-6.22)	5.75 (3007.94)	5.65 (5.26)	2.5 (2.38)
polyneuropathy	141	5.55 (4.7-6.56)	5.54 (514.54)	5.45 (4.61)	2.45 (2.16)
peripheral sensory neuropathy	101	7.93 (6.51-9.67)	7.92 (593.82)	7.73 (6.34)	2.95 (2.57)
peripheral motor neuropathy	44	14.44 (10.66-19.55)	14.43 (522.67)	13.76 (10.16)	3.78 (2.98)
guillain-barre syndrome	30	3.22 (2.25-4.62)	3.22 (45.41)	3.2 (2.23)	1.68 (1.06)
peripheral sensorimotor neuropathy	21	8.25 (5.35-12.74)	8.25 (129.89)	8.04 (5.21)	3.01 (1.98)
demyelinating polyneuropathy	9	3.95 (2.04-7.62)	3.94 (19.51)	3.9 (2.02)	1.96 (0.68)
acute polyneuropathy	5	7.23 (2.97-17.57)	7.23 (26.15)	7.07 (2.91)	2.82 (0.61)

Note: Sensitivity analysis was performed after excluding reports involving drugs known to cause peripheral neuropathy, including paclitaxel, docetaxel, nab-paclitaxel, cabazitaxel, cisplatin, carboplatin, oxaliplatin, vincristine, vinblastine, vinorelbine, bortezomib, and thalidomide.

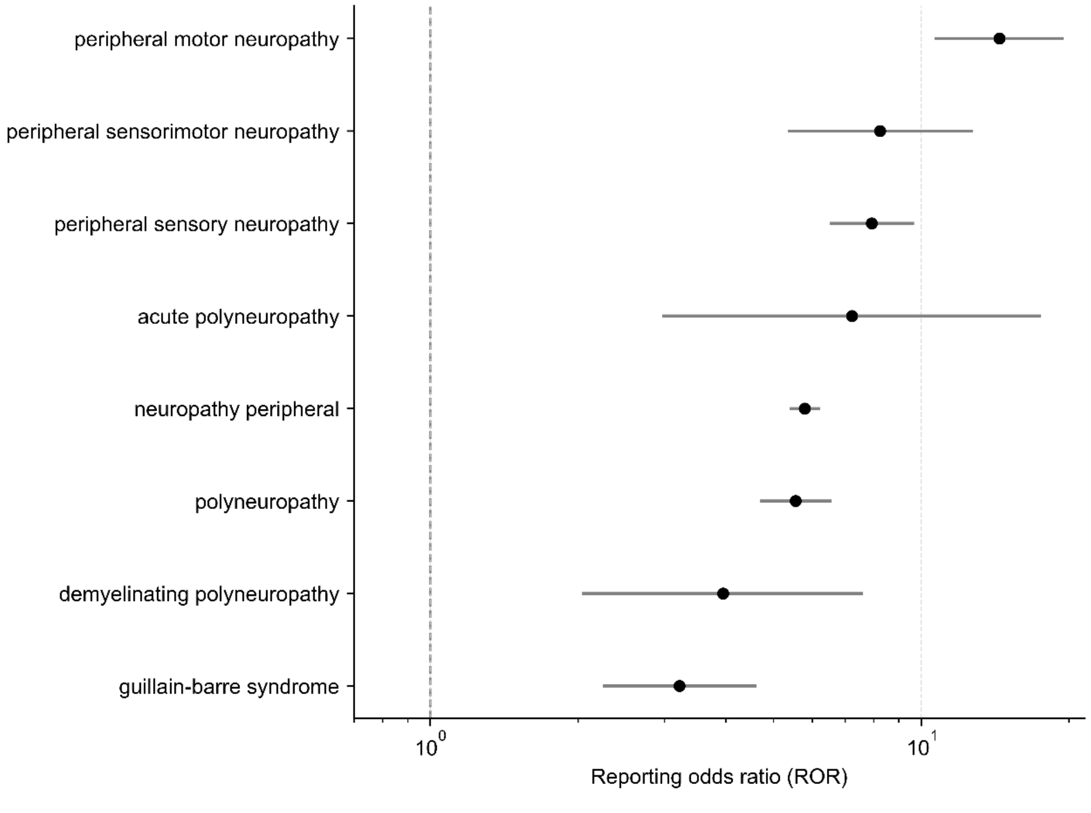

Supplementary Figure S1. Forest plot of disproportionality signals for peripheral nervous system (PNS) adverse events in the sensitivity analysis.

Sensitivity analysis was conducted by excluding reports involving drugs commonly associated with chemotherapy-induced peripheral neuropathy, including paclitaxel, docetaxel, nab-paclitaxel,

cabazitaxel, cisplatin, carboplatin, oxaliplatin, vincristine, vinblastine, vinorelbine, bortezomib, and thalidomide. Points represent reporting odds ratios (ROR), and horizontal lines indicate the 95% confidence intervals (95% CI). The vertical dashed line represents the null value (ROR = 1).
